# Beamforming Using Exact Evaluation of Leakage and Ergodic Capacity of MU-MIMO System

**DOI:** 10.3390/s21206792

**Published:** 2021-10-13

**Authors:** Ahmad Kamal Hassan, Muhammad Moinuddin

**Affiliations:** 1 Faculty of Electrical Engineering, GIK Institute of Engineering Sciences and Technology, Topi 23640, Pakistan; 2 Center of Excellence in Intelligent Engineering Systems (CEIES), King Abdulaziz University, Jeddah 21589, Saudi Arabia; mmsansari@kau.edu.sa; 3 Department of Electrical and Computer Engineering, King Abdulaziz University, Jeddah 21589, Saudi Arabia

**Keywords:** quadratic forms, Information theory, ergodic capacity, leakage rate, principal eigenvectors

## Abstract

Closed-form evaluation of key performance indicators (KPIs) of telecommunication networks help perform mathematical analysis under several network configurations. This paper deals with a recent mathematical approach of indefinite quadratic forms to propose simple albeit exact closed-form expressions of the expectation of two significant logarithmic functions. These functions formulate KPIs which include the ergodic capacity and leakage rate of multi-user multiple-input multiple-output (MU-MIMO) systems in Rayleigh fading channels. Our closed-form expressions are generic in nature and they characterize several network configurations under statistical channel state information availability. As a demonstrative example of the proposed characterization, the derived expressions are used in the statistical transmit beamformer design in a broadcast MU-MIMO system to portray promising diversity gains using standalone or joint maximization techniques of the ergodic capacity and leakage rate. The results presented are validated by Monte Carlo simulations.

## 1. Introduction

Quadratic forms find applications in multifarious fields of engineering. In telecommunication systems, quadratic forms in Gaussian random variables are of particular interest [1,2,3,4,5,6]. While the earlier works on quadratic forms in Gaussian random variables laid a good foundation, albeit a unified approach which can include cases of complex and real, central and non-central, and ratio formulation was not explored until recent work by Al-Naffouri et al. in [7]. That work provided several closed-form results of key performance indicators (KPI) of information theocratic systems such as outage probability. However, metrics dealing with the expectation of logarithmic functions such as ergodic capacity and leakage rate were not characterized therein. These metrics are central to any telecommunication network, and the availability of closed-form analytical formulas can lead to effective beamforming techniques.

Ergodic capacity provides an upper bound on reliable data rate over a fading channel. It is found by computing the expectations of a logarithmic function of signal-to-interference-plus-noise ratio (SINR). The SINR has a direct relationship with the ergodic capacity metric, whereas its cumulative distribution function (CDF) defines the outage probability measure. Furthermore, SINR expressions can account for both intra-cell as well as inter-cell interference [8]. Alternatively, signal-to-leakage-plus-noise ratio (SLNR) based metrics also directly or indirectly influence KPIs. Specifically, an intra-cell SLNR maximization problem is used for leakage interference suppression and outage analysis in [9], whereas, a sum leakage rate maximization problem is presented in [10] and its findings directly showcased as KPIs. Therefore, mathematical analysis of such KPIs can provide pedagogical insights into the design of multi-user multiple-input multiple-output (MU-MIMO) systems and other simpler models such as multiple-input single-output (MISO), and single-input single-output (SISO).

Various studies have been conducted to evaluate the ergodic capacity and related KPIs of communication systems. In what follows, we categorize different characterization approaches available in literature in terms of numerical solutions, exact solutions albeit with assumptions, and approximate solutions. Numerical solutions of the ergodic and outage capacity are presented in [11] in terms of a single integral formulation. Exact solutions, albeit with assumptions appear in [12,13,14,15,16] among other works. More specifically, a closed-form solution of maximum ratio combining (MRC) based systems is given in [12], however, it is pertinent for a noise-limited case. The work in [13] uses a moment generating function (MGF) based solution which is specific for non-negative random variables. Furthermore, refs. [14,15] consider bounds on the number of transmit antennas. A promising solution appears in [16], however, it is strictly for orthogonal beamforming vectors and requires channel state information (CSI) at the transmit side. Approximate solutions of KPIs include but are not limited to [17,18,19,20,21]. Therein, the solution in [17] is for large system analysis and it utilizes random matrix theory. The work in [18] considers a satellite to terminal node linked through a relay and the work accounts for outdated CSI, while [19] presents solutions by considering correlated channels under transmit antenna selection based scheme. An indefinite quadratic forms approach [7] is used to obtain an exact solution of the outage probability expression of a covariance shaping channel model in [22], however, only approximate solutions of ergodic capacity are reported under this approach. The approximate solutions include [20] which is based on a Joint-Diagonalization based approximation only suitable for commuting matrices and [21] which is relevant for large antenna arrays and specific to the orthonormal set of beamforming vectors. Hence, there is a need for a unified approach that provides exact closed-form expression of ergodic capacity by relaxing the above-mentioned assumptions on the transmit beamformers, antenna diversity, correlated channel gains, and indefinite weight matrices involved in SINR and SLNR expressions.

A common assumption found in literature is that the base station has knowledge of instantaneous CSI, e.g., [9,10,23]. These assumptions are based on a feedback scenario, wherein an accurate instantaneous CSI is made available at the base station by utilizing a significant fraction of the allocated bandwidth. Hence, as an alternate to the feedback path, a statistical CSI-based system is bandwidth-efficient and often more applicable in telecommunications. However, barring the simplest case of uncorrelated channels, deriving closed-form expressions of the network KPIs by considering only the channel statistics have difficult mathematical tractability even for Gaussian channels (e.g., [24]). Hence, deriving exact solutions of KPIs under statistical CSI availability is challenging and an active research area.

Motivated by the aforementioned discussions, we utilize the indefinite quadratic forms approach and contribute by:

1. deriving tractable, simple, and ‘exact’ closed-form solutions of the ergodic capacity and leakage rate. Our solutions relax the assumption of orthonormal and orthogonal transmit beamforming vectors, while they account for colored channels of arbitrary dimension, distinct correlation matrices, and indefinite eigenvalue structures. Furthermore, the proposed solutions are generic and applicable for any antenna diversity model,
2. demonstrating the derived closed-form expressions on a downlink broadcast MU-MIMO system. We also outline the design of transmitting beamformers by selecting sum-capacity and/or sum-leakage rate as objective functions and thereby employ a maximization problem under power loading constraints, and 
3. showcasing the efficacy of proposed results on several important works in the physical layer domain of communication systems.

Rest of this paper is organized as follows. Section 2 presents closed-form solutions of ergodic capacity and leakage rate alongside some special cases. Section 3 gives an application example, validates the main results, and outlines a beamformer design. Finally, Section 4 concludes the paper.

**Notations**: Vectors and matrices are indicated by bold letters. ${∥a∥}^{2}$ and |A| denote norm-2 of vector **a** and determinant of matrix **A**, respectively. E(.) represents the expectation operator and the expectations is with respect to all sources of randomness. CN(0,R) defines a zero-mean circularly-symmetric complex Gaussian distribution with covariance matrix **R**. ${\left(.\right)}^{H}$, u(.), and $E_{1}\left(.\right)$ indicate the conjugate transposition, the unit step function, and the exponential integral function, respectively. For brevity of notations ${\left(.\right)}^{\frac{H}{2}}$ is used to identify ${\left({\left(.\right)}^{\frac{1}{2}}\right)}^{H}$. For any matrix **A** and vector **h**, the quadratic form is defined as ${∥h∥}_{A}^{2}\overset{△}{\;=}h^{H}A\;h$.

## 2. The Main Results

In this section, we present theorems and some special cases thereof to evaluate ‘exact’ closed-form expression of the ergodic capacity and leakage rate. The two metrics are defined as follows:

(1) $$\begin{array}{ccc} C_{k} & = & E\left[log_{2}\left(1+\frac{h_{k}^{H}A\;h_{k}}{1+h_{k}^{H}B\;h_{k}}\right)\right], \end{array}$$

(2) $$\begin{array}{ccc} L_{k} & = & E\left[log_{2}\left(1+\frac{h_{k}^{H}A\;h_{k}}{1+h_{i}^{H}C\;h_{i}}\right)\right], \end{array}$$

where $C_{k}$ and $L_{k}$ represents the ergodic capacity and leakage rate of *k*th user equipment, respectively, while $h_{k}$ and $h_{i}$ are independent *k*th and *i*th Rayleigh fading channel vectors, respectively. Here, **A**, **B**, and **C** are indefinite Hermitian matrices which are assumed as deterministic.

**Theorem** **1.** *Given a channel vector $h_{k}∼\mathrm{CN}\left(0,R_{k}\right)$ of length T and indefinite Hermitian matrices* **A** *and* **B** *of dimension T×T. Then*

(3) $$\begin{array}{ccc} E\left[log_{2}\left(1+\frac{h_{k}^{H}A\;h_{k}}{1+h_{k}^{H}B\;h_{k}}\right)\right] & = & \frac{1}{\ln(2)}[\sum_{t=1}^{T}\frac{\lambda_{t}^{T-1}}{\prod_{u=1,u\neq t}^{T}\left(\lambda_{t}-\lambda_{u}\right)}e^{\frac{1}{\lambda_{t}}}E_{1}\left(\frac{1}{\lambda_{t}}\right)u\left(\lambda_{t}\right)\\ & & -\sum_{t=1}^{T}\frac{\nu_{t}^{T-1}}{\prod_{u=1,u\neq t}^{T}\left(\nu_{t}-\nu_{u}\right)}e^{\frac{1}{\nu_{t}}}E_{1}\left(\frac{1}{\nu_{t}}\right)u\left(\nu_{t}\right)], \end{array}$$

*where $\lambda_{t}$ and $\nu_{t}$ are the t-th eigenvalues of matrices $R_{k}^{\frac{1}{2}}\left[A+B\right]R_{k}^{\frac{H}{2}}$ and $R_{k}^{\frac{1}{2}}BR_{k}^{\frac{H}{2}}$, respectively.*

**Proof.**  The proof is given in Appendix A.   □

**Theorem** **2.** *Given two independent channel vectors $h_{k}∼\mathrm{CN}\left(0,R_{k}\right)$ and $h_{i}∼\mathrm{CN}\left(0,R_{i}\right)$ of length T and V, and indefinite Hermitian matrices* **A** *and* **C** *of dimension T×T and V×V, respectively. Then*

(4) $$\begin{array}{ccc} E\left[log_{2}\left(1+\frac{h_{k}^{H}A\;h_{k}}{1+h_{i}^{H}C\;h_{i}}\right)\right] & = & \frac{1}{\ln(2)}\sum_{t=1}^{T}\sum_{v=1}^{V}\frac{\kappa_{t}^{T}}{\prod_{\begin{array}{c} u=1,\\ u\neq t \end{array}}^{T}\left(\kappa_{t}-\kappa_{u}\right)}\frac{\varsigma_{v}^{V-1}}{\prod_{\begin{array}{c} w=1,\\ w\neq v \end{array}}^{V}\left(\varsigma_{v}-\varsigma_{w}\right)}\\ & & \times \frac{1}{\left(\kappa_{t}-\varsigma_{v}\right)}\left[e^{\frac{1}{\kappa_{t}}}E_{1}\left(\frac{1}{\kappa_{t}}\right)-e^{\frac{1}{\varsigma_{v}}}E_{1}\left(\frac{1}{\varsigma_{v}}\right)\right]u\left(\kappa_{t}\right)u\left(\varsigma_{v}\right),\;\;\;\;\; \end{array}$$

*where $\kappa_{t}$ and $\varsigma_{v}$ are the t-th and v-th eigenvalues of matrices $R_{k}^{\frac{1}{2}}AR_{k}^{\frac{H}{2}}$ and $R_{i}^{\frac{1}{2}}CR_{i}^{\frac{H}{2}}$, respectively.*

**Proof.**  The proof is given in Appendix B.    □

Note that the two theorems cater for both the positive and negative eigenvalues. Herein, we only consider distinct eigenvalues, an extension for repetitive eigenvalues is simple and for such a differential approach in [25] is useful. The theorems include the SINR and SLNR and they are applicable on the systems with transmit and receive antenna diversity. The quadratic forms for such systems is pointed out in [22], furthermore [22,26] also provide efficient statistical beamforming vector designs. Statistics of channel vectors indicate that such beamforming vector designs are in the direction of the principal eigenvector (PEV) of the channel correlation matrices [27].

The expectation of the form involving signal-to-noise ratio (SNR) is rather mature in literate. An ‘exact’ solution for the noise-limited case is established [20] for unit rank matrices. In Proposition 1, we provide a solution for an arbitrary rank Hermitian matrices under the framework of indefinite quadratic forms.

**Proposition** **1.** *As a special case of Theorem 1, considering weight matrix* **B** *=* **0** *. Then:*

(5) $$\begin{array}{c} E\left[log_{2}\left(1+h_{k}^{H}A\;h_{k}\right)\right]=\frac{1}{\ln(2)}\left[\sum_{t=1}^{T}\frac{\kappa_{t}^{T-1}}{\prod_{u=1,u\neq t}^{T}\left(\kappa_{t}-\kappa_{u}\right)}e^{\frac{1}{\kappa_{t}}}E_{1}\left(\frac{1}{\kappa_{t}}\right)u\left(\kappa_{t}\right)\right]. \end{array}$$

**Proof.**  The proof is pointed out in Appendix A.   □

The aforementioned proposition is useful in deriving the expectations of the form [28] ((5)–(7)) by interweaving sum of quadratic forms, i.e., $h_{k}^{H}A\;h_{k}=h_{k}^{H}\left\{\sum_{c=1}^{C}A_{c}\right\}\;h_{k}=h_{k}^{H}A_{1}\;h_{k}+h_{k}^{H}A_{2}\;h_{k}+\cdots +h_{k}^{H}A_{C}\;h_{k}$. With some manipulations, Proposition 1 can also be applied on cognitive radio and relay-assisted systems with partial channel state information [29] (8)–[30] (20).

Next, a structure pertinent to the communication systems having white or identical correlation matrices is proposed.

**Proposition** **2.** *As a special case of Theorem 2, considering white channels, i.e.,  $h_{k}∼\mathrm{CN}\left(0,I\right)$, $h_{i}∼\mathrm{CN}\left(0,I\right)$, and assuming similar weight matrices, i.e., ***A** = **C** *. Then:*

(6) $$\begin{array}{c} E\left[log_{2}\left(1+\frac{h_{k}^{H}A\;h_{k}}{1+h_{i}^{H}A\;h_{i}}\right)\right]=\frac{1}{\ln(2)}\sum_{t=1}^{T}\frac{\kappa_{t}^{2T-3}}{\prod_{\begin{array}{c} u=1,\\ u\neq t \end{array}}^{T}{\left(\kappa_{t}-\kappa_{u}\right)}^{2}}\left[\kappa_{t}-e^{\frac{1}{\kappa_{t}}}E_{1}\left(\frac{1}{\kappa_{t}}\right)\right]u\left(\kappa_{t}\right), \end{array}$$

*where $\kappa_{t}$ is now the tth eigenvalue of matrix A since $R_{k}=I$.*

**Proof.**  The proof is pointed out in Appendix B.    □

Proposition 2 provides statistical insights of leakage rate defined similarly in [10] (4).

## 3. Application Example and Discussion

**Figure 1:**
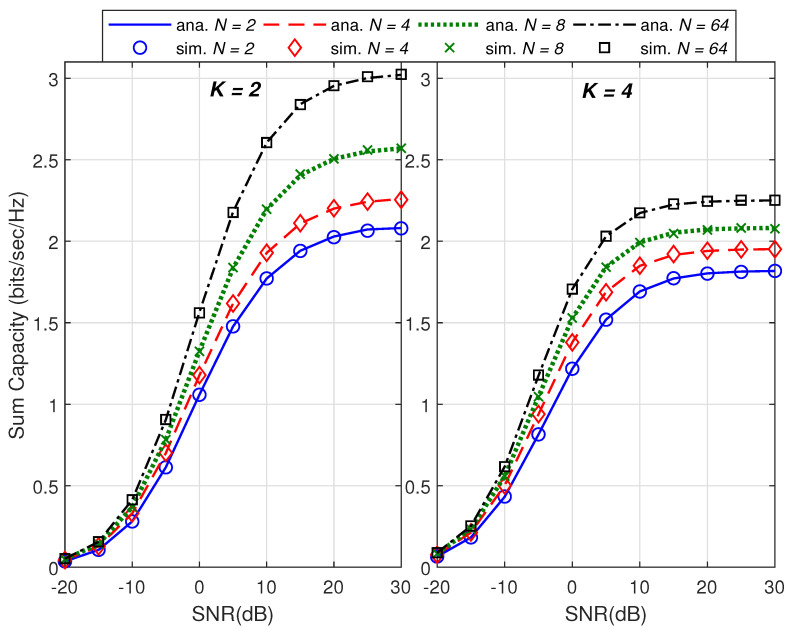
Comparison of analytical and simulation results of the sum capacity versus the transmit SNR in dB.

In this section, we demonstrate the utility of the proposed theorems by adopting the broadcast system model given in [20,31] (Figure 1). Specifically, we deal with a downlink single cell MU-MIMO system consisting of *K* single antenna mobile stations (MS) and an *N* antenna base station (BS). We assume unity average power symbols which are modulated with transmit beamformer $w_{k}\in C^{N\times 1}$ at transmission end, sent over an N×1 channel $h_{k}∼\mathrm{CN}\left(0,R_{k}\right)$, and received at the *k*th MS. The system is also inflicted with an additive white noise with zero mean and variance $\sigma_{k}^{2}$. Hence, the instantaneous SINR and SLNR of the *k*th user under this model is given by

(7) $$\begin{array}{ccc} {\mathrm{SINR}}_{k} & = & \frac{|h_{k}^{H}w_{k}{|}^{2}}{\sigma_{k}^{2}+\sum_{i=1,i\neq k}^{K}{\left|h_{k}^{H}w_{i}\right|}^{2}}, \end{array}$$

(8) $$\begin{array}{ccc} {\mathrm{SLNR}}_{k} & = & \frac{|h_{k}^{H}w_{k}{|}^{2}}{\sigma_{k}^{2}+\sum_{i=1,i\neq k}^{K}{\left|h_{i}^{H}w_{k}\right|}^{2}}, \end{array}$$

where $w_{i}$ represents the beamforming vector of interfering symbol and $h_{i}∼\mathrm{CN}\left(0,R_{i}\right)$ represents the leakage channel.

Now, employing the whitening transformations on the desired and interference channels, i.e., ${\bar{h}}_{k}=R_{k}^{-\frac{H}{2}}h_{k}$, and ${\bar{h}}_{i}=R_{i}^{-\frac{H}{2}}h_{i}$, respectively, allows us to express SINR*_k_* and SLNR*_k_* in the canonical quadratic form appearing in the proposed theorems as:

(9) $$\begin{array}{ccc} {\mathrm{SINR}}_{k} & = & \frac{{\bar{h}}_{k}^{H}A{\bar{h}}_{k}}{1+{\bar{h}}_{k}^{H}B{\bar{h}}_{k}}; \end{array}$$

(10) $$\begin{array}{ccc} {\mathrm{SLNR}}_{k} & = & \frac{{\bar{h}}_{k}^{H}A{\bar{h}}_{k}}{1+{\bar{h}}_{i}^{H}C{\bar{h}}_{i}}, \end{array}$$

where **A**, **B**, and **C** are the weight matrices of the desired, co-channel interference, and leakage interference, namely

$$\begin{array}{c} A=\frac{1}{\sigma_{k}^{2}}R_{k}^{\frac{1}{2}}w_{k}w_{k}^{H}R_{k}^{\frac{H}{2}},\;\;B=\frac{1}{\sigma_{k}^{2}}R_{k}^{\frac{1}{2}}\left(\sum_{i=1,i\neq k}^{K}w_{i}w_{i}^{H}\right)R_{k}^{\frac{H}{2}},\;\;\mathrm{and}\;\;C=\frac{1}{\sigma_{k}^{2}}R_{i}^{\frac{1}{2}}w_{k}w_{k}^{H}R_{i}^{\frac{H}{2}}. \end{array}$$

Next, considering that receiver has channel state information (CSI), the transmitter has statistical CSI, the channels are ergodic, and bandwidth is normalized to unity; then the sum ergodic capacity *C* [32], and the sum leakage rate *L* is expressed as

(11) $$\begin{array}{ccc} C & = & \sum_{k=1}^{K}C_{k}=\sum_{k=1}^{K}E\left[log_{2}\left(1+{\mathrm{SINR}}_{k}\right)\right], \end{array}$$

(12) $$\begin{array}{ccc} L & = & \sum_{k=1}^{K}L_{k}=\sum_{k=1}^{K}E\left[log_{2}\left(1+{\mathrm{SLNR}}_{k}\right)\right], \end{array}$$

where $C_{k}$ and $L_{k}$ have now simple and exact closed-forms thanks to (3) and (4), respectively.

### 3.1. Validation of the Closed-Form Expressions

**Figure 2:**
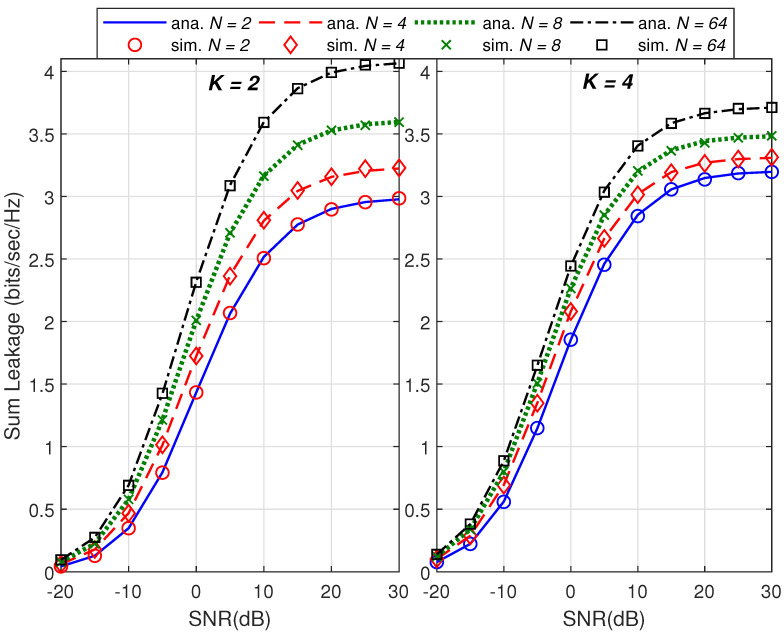
Comparison of analytical and simulation results of the sum leakage rate versus the transmit SNR in dB.

**Figure 3:**
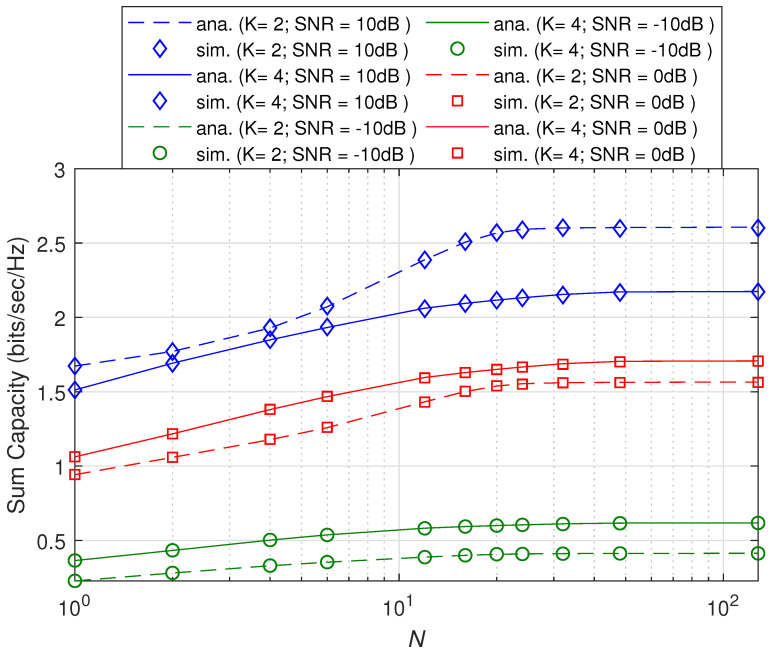
Comparison of analytical and simulation results of the sum capacity rate versus the transmit antenna order *N* in log scale.

**Figure 4:**
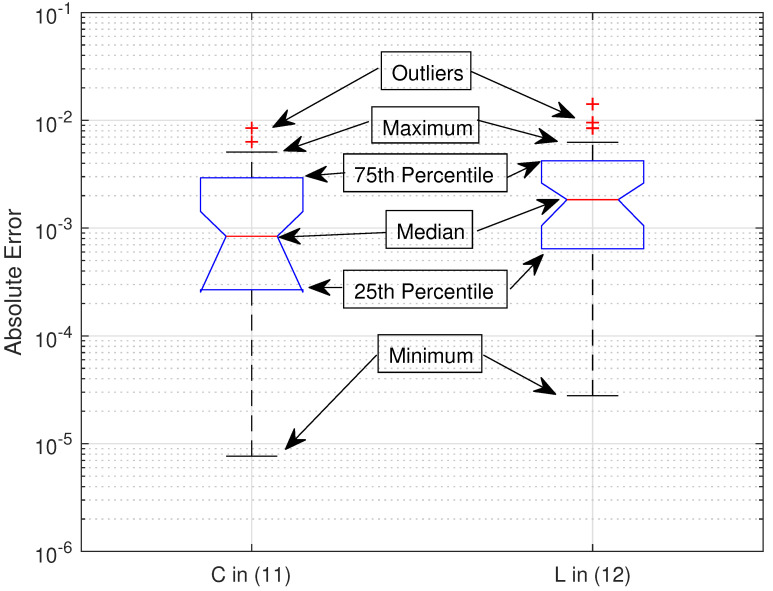
Summary statistics of absolute error between analytical and simulation results of the sum capacity and sum leakage rate. The outliers denoted by ‘+’ marker indicate the values which fall 1.5 times away from the top interquartile range of the box plots.

For validation purpose of the proposed work, we show in Figure 1 and Figure 2 the plots of the sum capacity and sum leakage rate against SNR in dB while varying the number of transmit antennas *N*. Herein, we consider distinct correlation matrices and set the Monte Carlo runs to 100,000. With insight from [27], transmit beamformers, i.e., $w_{k},\;\forall k$ are based on the principal eigenvector of the respective correlation matrices. Note that the system behaves as noise limited at low SNR and interference limited at high SNR as observed for *K* = 2 and *K* = 4 in both the figures. We have used a dominant eigenvalue-based beamformer design [27] in the simulation setup which is not optimal and hence degradation in sum capacity and leakage is observed as the total number of users increase. However for large *K*, it is observed that the rate degradation in Figure 2 is less than Figure 1 because the leakage rate in (11) is a function of decoupled beamformers (8). An increase in transmit antenna order *N* also increases beam directivity and hence increases sum and leakage rates in both the figures. In Figure 3, plot of the sum capacity against an increasing *N* while varying SNR in dB is given. For the PEV beamformer design, the performance gains slow down with the increasing number of transmit antennas for all considered cases. The absolute error between the analytical closed-form expressions and simulations of 100,000 Monte Carlo runs is presented in Figure 4. Note that the summary statistics would further improve by increasing the Monte Carlo runs. Hence, under several network configurations including the case for large array MIMO structures, an exact match of analytical and simulation results proves the two theorems.

### 3.2. Beamforming Using the Closed-Form Expressions

The availability of the closed-form expressions allows us to utilize state-of-art unsupervised transmit antenna beamformer designs. The design is unsupervised in the sense that it considers only the statistical CSI at the transmitter side. As *C* and *L* given in (11) and (12) account for all users in the respective expressions, a single objective optimization problem is desired. We define such optimization problem as

(13) $$\begin{array}{cccc} \; & \max_{{\left\{w_{k}\right\}}_{k=1}^{K}}\; & \; & J\left({\left\{w_{k}\right\}}_{k=1}^{K};C;L\right)\\ \; & \;\;s.t. & \; & {\left\{{∥w_{k}∥}_{2}^{2}\leq 1\right\}}_{k=1}^{K}\;, \end{array}$$

where the constraints are used to limit transmit power of all transmit beamformers, while depending on the methodology of optimization; the objective function has the following cases

(14) $$\begin{array}{c} J\left(.\right)=\left\{\begin{array}{cc} L, & \mathrm{Standalone}\;L\;\max.\;\\ C, & \mathrm{Standalone}\;C\;\max.\;\\ C↔L, & \;\mathrm{Joint}\;C\;\mathrm{and}\;L\;\max. \end{array}\right. \end{array}$$

In the standalone case, either *L* or *C* is used as an objective function. In the joint case, both C↔L are set as objectives in the alternate recursions. Hence, after initialization using the PEV approach [27] in the joint case, (11) is selected in (13) at odd recursion and the results obtained are then used as initial beam vectors for (12) which are now used at even recursions in (13). The algorithm terminates if the relative improvement of (13) is less than the predefined threshold value set by the user. The working principle of (13) and (14) is based on the ‘interior-point’ method given in [33]. The construct of the beamformer is given in Algorithm 1.
**Algorithm 1** Construct of Beamformer1:Set the iteration index i=0, and define algorithm termination conditions, namely, maximum iterations ($i_{max}$), and precision level (ϵ).2:Select an objective function from (14), and initialize beamformers ${\left\{w_{k}^{\left\{int.\right\}}\right\}}_{k=1}^{K}$ using PEV scheme in [27].3:Compute $J^{\left(i\right)}\left({\left\{w_{k}\right\}}_{k=1}^{K};C;L\right)$ using ${\left\{w_{k}^{\left\{int.\right\}}\right\}}_{k=1}^{K}$.4:**repeat**5:    i=i+16:    Compute $J^{\left(i\right)}\left({\left\{w_{k}\right\}}_{k=1}^{K};C;L\right)$ using ‘interior-point’ method in (13).7:    Update local optimal beamformer ${\left\{w_{k}^{lo}\right\}}_{k=1}^{K}$.8:    **if** $\left\{\right|J^{\left(i\right)}\left(.\right)-J^{\left(i-1\right)}\left(.\right)\left|\;\geq \;\epsilon \right\}\;\;\&\&\;\;\left\{i_{}\leq i_{max}\right\}$ **then**9:        **set**  Condition = false.10:    **else**11:        **set  **Condition = true.12:    **end if**13:**until** {Condition = true}

**Figure 5:**
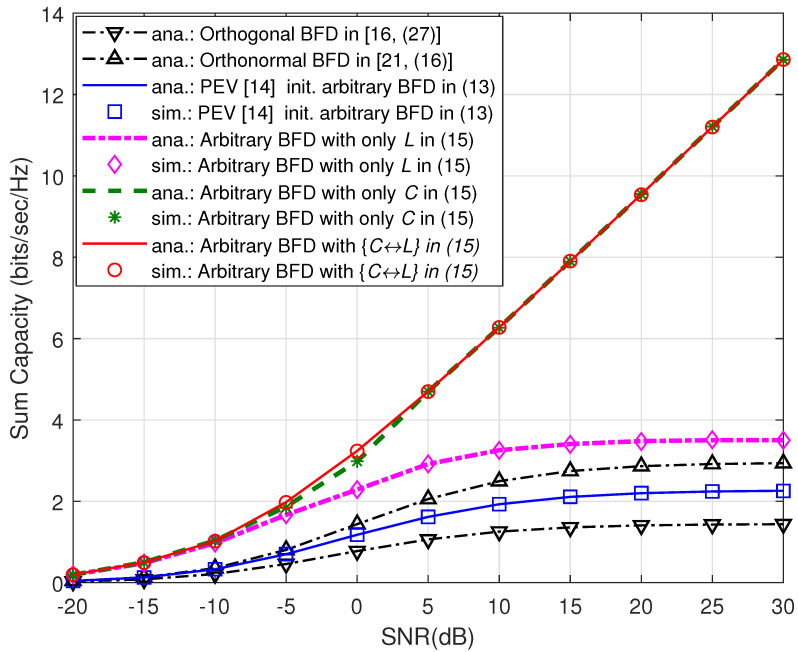
Comparison of analytical and simulation results of the sum capacity versus the transmit SNR in dB under several beamformer designs (BFD).

In Figure 5, we reflect on the aforementioned optimization problem by setting *N* = 4 and *K* = 2, while performing initialization of transmitting beamformers using PEV. Performance of the three cases in (14) is compared in terms of the sum capacity versus SNR in dB. Under the considered network configurations, Joint C↔L max. is most efficient, Standalone *C* max. is finding good local maxima, whereas Standalone *L* max. is inefficient at high SNR values. In Figure 5, we also provide the validity of the proposed theorems at both initialization and post-optimization phases. In comparison, the solutions in [16] which is specific for orthogonal beamformers, and [21] which is based on the orthonormal set of beamformers fails validation under the PEV initialized arbitrary beamformer designs.

**Figure 6:**
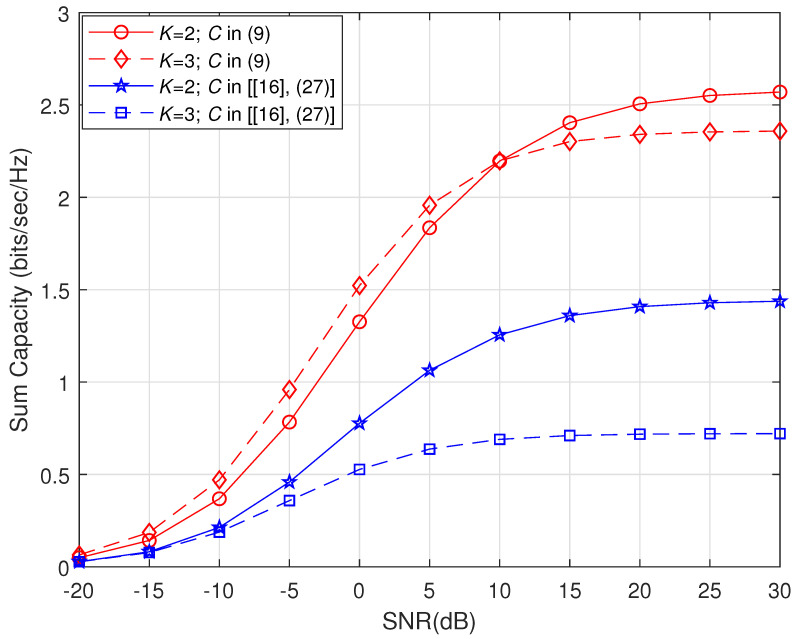
Comparison of the sum capacity versus the transmit SNR in dB. Here, proposed and existing solutions are based on the PEV transmit beamforming scheme.

Lastly, in Figure 6, the performance of the proposed solution is compared with [16] (27) for *K* set to 2 and 3. It is worth noting that the compared closed-form solution is based on known CSI at the transmit side and their model considers channel knowledge at the transmit side and uses it in the transmit beamformer design. However, for a fair comparison, we subject their solution to the PEV based arbitrary beamformer design owing to only statistical CSI availability at the transmit side. It is observed that until the SNR value of 10 dB, the proposed solution behaves as noise limited whereas, beyond this value, the system is more interference impaired. Nevertheless, the proposed solutions in both the user cases show significantly higher performance throughout the SNR range as compared with the exiting method.

## 4. Conclusions

In this paper, exact closed-form expressions of the ergodic capacity and leakage are presented for a generic model which includes co-channel interference, distinct correlation matrices, Hermitian indefinite weight matrices of correlated channels, and any deterministic beamformers. The expressions are in terms of exponential, exponential integral, and unit step functions. In the demonstration, a downlink broadcast MU-MIMO system impaired by Rayleigh fading is considered, and a canonical SINR and SLNR formulation is outlined on which the proposed solution can be directly applied. Furthermore, the proposed design of transmitting beamformers indicates that KPIs of telecommunication systems can be enhanced both by using SINR and SLNR based metrics. The proposed results hold not only for classical network configurations but also for emerging large antenna array models where transmit antenna elements are greater than the total number of users in a given cell. This work can be furthered in several domains including the performance analysis of covariance shaping receive beamformers, characterization of multi-cell association systems, and performance optimization of cognitive radio and relay assisted networks.

## Data Availability

Not applicable.

## Appendix A

**Proof** **of** **Theorem 1.** From the interweaving of sum of quadratic forms defined in Section 2, employing the notion of quadratic forms, and the linearity of the expectation operator, we have

(A1) $$\begin{array}{c} C_{k}=\underset{I_{1}}{\underset{︸}{E\left[log_{2}\left(1+\left|\right|h_{k}{\left|\right|}_{A+B}^{2}\right)\right]}}-\underset{I_{2}}{\underset{︸}{E\left[log_{2}\left(1+\left|\right|h_{k}{\left|\right|}_{B}^{2}\right)\right]}} \end{array}$$

Using [34] (6), $I_{1}$ is expressed in integral form as follows

(A2) $$\begin{array}{ccc} I_{1} & = & \int_{0}^{\infty }\log_{2}\left(1+\gamma_{th}\right)\;f_{\left|\right|h_{k}{\left|\right|}_{A+B}^{2}}\left(\gamma_{th}\right)\;d\gamma_{th} \end{array}$$

(A3) $$\begin{array}{ccc} & = & \frac{1}{\ln(2)}\int_{0}^{\infty }\frac{1-F_{\left|\right|h_{k}{\left|\right|}_{A+B}^{2}}\left(\gamma_{th}\right)}{\gamma_{th}+1}d\gamma_{th} \end{array}$$

where $\gamma_{th}$ is a predefined threshold of the CDF, i.e., $F_{\left|\right|h_{k}{\left|\right|}_{A+B}^{2}}\left(\gamma_{th}\right)=\mathrm{Pr}\{||h_{k}{\left|\right|}_{A+B}^{2}<\gamma_{\mathrm{th}}\}$, and $f_{\left|\right|h_{k}{\left|\right|}_{A+B}^{2}}\left(\gamma_{th}\right)$ is the probability density function (PDF). The second equality is obtained by using integration by parts [35] (20). Now, applying residue theory [7], the closed-form expression of the CDF is expressed as

(A4) $$F_{\left|\right|h_{k}{\left|\right|}_{A+B}^{2}}\left(\gamma_{th}\right)=1-\sum_{t=1}^{T}\frac{\lambda_{t}^{T-1}e^{-\frac{\gamma_{th}}{\lambda_{t}}}}{\prod_{u=1,u\neq t}^{T}\left(\lambda_{t}-\lambda_{u}\right)}u\left(\lambda_{t}\right)$$

Next, plugging (A4) in (A2), applying change of variable ${\bar{\gamma }}_{th}=\gamma_{th}+1$, and employing [36] (Proposition 3.351-5), gives

(A5) $$I_{1}=\frac{1}{\ln(2)}\sum_{t=1}^{T}\frac{\lambda_{t}^{T-1}}{\prod_{u=1,u\neq t}^{T}\left(\lambda_{t}-\lambda_{u}\right)}e^{\frac{1}{\lambda_{t}}}E_{1}\left(\frac{1}{\lambda_{t}}\right)u\left(\lambda_{t}\right)$$

The second integral $I_{2}$ in (A1) has **B** as the weight matrix of the quadratic norm and has a similar structure differing only by the eigenvalues. Hence, the closed-form expressions of $I_{1}$ and $I_{2}$ yield the exact solution given in (3). The same methodology applies to obtain the latest expression in (5). □

## Appendix B

**Proof** **of** **Theorem 2.** The proof starts by conditioning the channel vector appearing in the denominator in (4) as

(A6) $$L_{k}{|}_{\left|\right|h_{i}{\left|\right|}_{C}^{2}=x}=E\left[log_{2}\left(1+\left|\right|h_{k}{\left|\right|}_{A/(1+x)}^{2}\right)\right]$$

Using the integral formulation shown in the proof of Theorem 1 and removing the condition in (A6) gives us

(A7) $$\begin{array}{c} L_{k}=\frac{1}{\ln(2)}\int_{-\infty }^{\infty }\int_{0}^{\infty }\frac{1-F_{\left|\right|h_{k}{\left|\right|}_{A/(1+x)}^{2}}\left(\gamma_{th}\right)}{\gamma_{th}+1}f_{\left|\right|h_{i}{\left|\right|}_{C}^{2}}\left(x\right)d\gamma_{th}dx \end{array}$$

where CDF and PDF are characterized using [7] as follows

(A8) $$\begin{array}{ccc} F_{\left|\right|h_{k}{\left|\right|}_{A/(1+x)}^{2}}\left(\gamma_{th}\right) & = & \mathrm{Pr}\{||h_{k}{\left|\right|}_{A/(1+x)}^{2}<\gamma_{\mathrm{th}}\}\;\;\;\;\;\;\;\;\;\;\;\;\;\;\\ & = & 1-\sum_{t=1}^{T}\frac{\kappa_{t}^{T-1}}{\prod_{\begin{array}{c} u=1,\\ u\neq t \end{array}}^{T}\left(\kappa_{t}-\kappa_{u}\right)}e^{-\frac{\left(1+x\right)\gamma_{th}}{\kappa_{t}}}u\left(\frac{\left(1+x\right)\gamma_{th}}{\kappa_{t}}\right) \end{array}$$

(A9) $$\begin{array}{ccc} f_{\left|\right|h_{i}{\left|\right|}_{C}^{2}}\left(x\right) & = & \frac{d}{dx}\mathrm{Pr}\{||h_{i}{\left|\right|}_{C}^{2}<x\}\;\;\;\;\;\;\;\;\;\;\;\;\;\;\;\;\;\;\;\;\;\;\;\;\;\;\;\;\;\;\\ & = & \sum_{v=1}^{V}\frac{\varsigma_{v}^{V-2}}{\prod_{w=1,w\neq v}^{V}\left(\varsigma_{v}-\varsigma_{w}\right)}e^{-\frac{x}{\varsigma_{v}}}u\left(\frac{x}{\varsigma_{v}}\right)\;\;\;\;\;\;\; \end{array}$$

Next, by plugging (A8) and (A9) in (A7), adjusting the limits of integration by means of the two-unit step functions, and performing some mathematical manipulations yield

(A10) $$\begin{array}{ccc} L_{k} & = & \sum_{t=1}^{T}\sum_{v=1}^{V}\frac{\kappa_{t}^{T}e^{\frac{1}{\kappa_{t}}}u\left(\kappa_{t}\right)}{\prod_{u=1,u\neq t}^{T}\left(\kappa_{t}-\kappa_{u}\right)}\frac{\varsigma_{v}^{V-1}u\left(\varsigma_{v}\right)}{\prod_{w=1,w\neq v}^{V}\left(\varsigma_{v}-\varsigma_{w}\right)}\\ & \times & \frac{1}{\ln(2)}\left[\int_{1}^{\infty }\frac{1}{\gamma_{th}\left(\gamma_{th}+\frac{\kappa_{t}}{\varsigma_{v}}-1\right)}e^{\frac{\gamma_{th}}{\kappa_{t}}}d\gamma_{th}\right] \end{array}$$

By partial fraction expansion of (A10) and using [36] (Propositions 3.351-5 and 3.352-2) leads to the solution given in (4). For the special case in (6), we consider **A** = **C** and hence use $f_{\left|\right|h_{i}{\left|\right|}_{A}^{2}}\left(x\right)$ in (A7) followed by some mathematical manipulations, to achieve a single integral form as

(A11) $$\begin{array}{ccc} L_{k} & = & \frac{1}{\ln(2)}\sum_{t=1}^{T}\frac{\kappa_{t}^{2(T-1)}}{\prod_{u=1,u\neq t}^{T}{\left(\kappa_{t}-\kappa_{u}\right)}^{2}}u\left(\kappa_{t}\right)\int_{0}^{\infty }\frac{1}{{\left(\gamma_{th}+1\right)}^{2}}e^{\frac{\gamma_{th}+1}{\kappa_{t}}}d\gamma_{th} \end{array}$$

Lastly, using [36] (Proposition 3.353-3) on the above expression yields the exact solution given in (6). □
